# Presence and type of decompensation affects outcomes in autoimmune hepatitis upon treatment with corticosteroids

**DOI:** 10.1002/jgh3.12451

**Published:** 2020-11-13

**Authors:** Sanchit Sharma, Samagra Agarwal, Kanav Kaushal, Abhinav Anand, Deepak Gunjan, Rajni Yadav, Anoop Saraya

**Affiliations:** ^1^ Department of Gastroenterology and Human Nutrition All India Institute of Medical Sciences New Delhi India; ^2^ Department of Pathology All India Institute of Medical Sciences New Delhi India

**Keywords:** ascites, autoimmune hepatitis, portal hypertension

## Abstract

**Background and Aims:**

Decompensated cirrhosis in autoimmune hepatitis has poor prognosis. Besides liver transplant, treatment for this entity is undefined. We explored the outcomes of autoimmune hepatitis (AIH)‐related decompensated cirrhosis with active disease on treatment with steroids.

**Methods:**

In this retrospective analysis, clinical data, laboratory parameters, and prognostic scores, such as baseline model for end‐stage liver disease (MELD) scores, were compared among patients of AIH with decompensated cirrhosis with mild/no ascites (*n* = 38), gross ascites (*n* = 24), and compensated cirrhosis (*n* = 32) when administered steroids. The primary outcome was transplant‐free survival at 12 months. Biochemical remission rates and other adverse events were also assessed and compared between these groups.

**Results:**

Steroids were initiated at lower doses (25 mg/day‐mild/no ascites, 20 mg/day‐gross ascites) in patients with decompensated cirrhosis and at 40 mg/day in those with compensated cirrhosis. Transplant‐free survival was 25.4%, 74.6%, and 96.9% (*P* = 0.001), and biochemical remission occurred in 5.1%, 49.0%, and 64.1% (*P* = 0.001) at 12 months in patients with gross ascites, mild/no ascites, and compensated cirrhosis, respectively. Infections were seen more frequently in decompensated cirrhosis, while other adverse events were comparable. Among decompensated cirrhosis, those with mild/no ascites had better prognostic scores, fewer posttreatment infections, and more frequent biochemical remission than those with gross ascites, achieving rates comparable to compensated cirrhosis. On multivariate analysis, the MELD score (subdistributional hazards ratio [sHR]; 95% confidence interval: 1.153 [1.07–1.24]; *P* = 0.001) and ascites (sHR: 2.556 [1.565–5.65]; *P* = 0.020) predicted survival.

**Conclusion:**

Type and severity of decompensation affect outcomes in patients with AIH‐related cirrhosis. Those with mild/no ascites have comparable outcomes to those with compensated cirrhosis upon treatment with low‐dose steroids.

## Graphical abstract

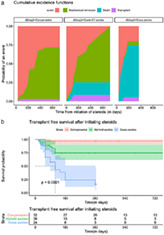

A subset of autoimmune hepatitis‐associated decompensated cirrhosis with only mild ascites/isolated variceal bleed achieves comparable biochemical remission to those having compensated cirrhosis when treated with steroids.

## Introduction

Cirrhosis in autoimmune hepatitis (AIH) is observed in about one‐third of patients at presentation and in 10–40% of patients on follow‐up.^1^, ^2^, ^3^, ^4^, ^5^, ^6^ The presence of cirrhosis portends a poorer response, increased risks of adverse events with treatment, and decreased survival in AIH.^1^, ^5^, ^7^, ^8^, ^9^, ^10^, ^11^ Despite poorer outcomes, however, treatment has been associated with the reversal of fibrosis in patients with compensated cirrhosis.^12^, ^13^, ^14^, ^15^ Hence, most international societies recommend treating active disease in AIH with compensated cirrhosis.^16^, ^17^

A subset of patients with AIH and cirrhosis have decompensated disease at presentation.^18^, ^19^, ^20^, ^21^ Advanced disease, intolerable adverse events with drugs, and increased risk of infections often preclude immunosuppression in these patients.^7^, ^16^, ^22^ At present, most societies limit their recommendations to the management of decompensations, as for other etiologies, rather than treatment of AIH per se.^16^, ^17^, ^23^ Management of decompensated cirrhosis thus represents an unmet need in the therapeutic paradigm of AIH, particularly in this part of the world where there is a larger subset of patients with AIH with decompensated cirrhosis and limited options for liver transplant.

The present study explored the treatment outcomes and adverse events with steroids in patients with AIH‐related decompensated cirrhosis with active disease at presentation when further stratified by the type and severity of decompensating events. The outcomes were compared with those of patients having compensated cirrhosis.

## Methods

### *Study design*

This study was conducted at a tertiary care center in northern India during the period of January 2014 to September 2019. From a prospectively maintained database, all patients of AIH (more than 14 years of age) with a pretreatment International Autoimmune Hepatitis Group (IAIHG) diagnostic criteria score of >10 (probable AIH) with evidence of cirrhosis were screened for inclusion. Patients with AIH: (i) who were previously treated with immunosuppression and had decompensated over follow‐up; (ii) having overlap with primary biliary cirrhosis (PBC) and/or primary sclerosing cholangitis (PSC); (iii) presenting with inactive disease; (iv) having active disease with decompensation but not given steroids; and (v) presenting with overt hepatic encephalopathy (HE) and acute‐on‐chronic liver failure (ACLF)^24^ were excluded from the primary analysis. For the present study, only those patients with AIH‐related cirrhosis with active disease (defined below) who received immunosuppression were included. All procedures performed in the studies were in accordance with the ethical standards of the institutional ethics committee and are in accordance with the 1964 Helsinki declaration and its later amendments or comparable ethical standards.

### *Risk groups*

For the present study, we subcategorized patients with decompensated cirrhosis at presentation into those with Grade 2/3 ascites^23^ (gross ascites) with or without variceal bleed at presentation and those with mild (Grade 1) ascites or no ascites (presentation with variceal bleed as the only decompensating event) to assess the impact of the type and severity of a decompensating event in determining the outcome. We chose ascites as the stratification event as it is the most important and usually the first decompensation in the natural history of cirrhosis.^25^ Another group of patients with compensated cirrhosis with active disease treated with immunosuppression was included, with which the outcomes in the decompensated cirrhosis cohort were compared.

### *Definitions*

AIH was diagnosed based on a constellation of clinical presentation, serology, and biopsy features suggestive of AIH. Cirrhosis was diagnosed based on the presence of >F4 fibrosis using the Ishak staging system^26^ on liver biopsy or clinical/endoscopic evidence of portal hypertension.^23^ Active disease was suspected if patients had any of the following: elevated transaminase levels (more than thrice the upper limit normal), elevated serum immunoglobulin G (IgG) levels of more than twice the upper limit normal,^16^, ^22^ and/or histological activity index (HAI) ≥4 on liver biopsy (using Ishak score).^26^ As patients with AIH with cirrhosis may have normal transaminase levels despite active disease and patients with cirrhosis may have elevated IgG levels^27^ even in absence of active disease, demonstration of HAI≥4 on liver biopsy was used as an essential criterion for active disease. Decompensated cirrhosis was diagnosed based on the presence of clinical decompensating events such as ascites or variceal bleed at the time of diagnosis.^23^ For treatment‐related outcomes, we assessed biochemical remission, progression of disease, and transplant‐free survival at 1 year. Biochemical remission was defined as complete normalization of transaminase levels in patients with previously elevated levels^17^ and was assessed at 6–12 months after treatment initiation. Progression of disease was arbitrarily chosen to be defined as worsening of Child Turcot Pugh (CTP) and MELD scores on follow‐up visit/or new‐onset decompensation in a patient or worsening of existing decompensations like progression of ascites. Transplant‐free survival was assessed by the proportion of patients surviving without transplant at defined time intervals.

### *Data collection*

Data collection is summarized in detail in Appendix S1 (Supporting information).

### *Treatment strategy*

Prior to the initiation of steroids, all patients were counseled about the expected benefits and adverse events of steroids/other immunosuppression. Metabolic screening for diabetes and blood pressure was conducted, and they were managed appropriately if present prior to the institution of treatment. All patients underwent bone mineral density assessment for screening of osteoporosis. Elemental calcium of 1000 mg/day and a weekly dose of vitamin D of 60000 units was given in all patients along with steroids. In addition, a detailed psychiatric assessment was performed by an experienced treating physician prior to the institution of steroids.

#### *Compensated cirrhosis*

Patients were initiated on a dose of prednisolone of 0.5–1 mg/kg (30–50 mg/day) once a day. Azathioprine was initiated at a dose of 1 mg/kg (if tolerated, titrated up to 2 mg/kg) 2 weeks after the initiation of steroids. Patients were initially assessed at 2‐week intervals, and steroids were tapered by 5 mg every 2 weeks after 4 weeks of the above dose if biochemical response was present. Steroids were tapered to the lowest dose required to maintain the response. For azathioprine, 2‐week blood counts were carried out till the desired dose was achieved, and thereafter, counts were repeated after every 4 weeks. In patients not achieving biochemical response while being compliant or in patients with intolerance, second‐line drugs such as mycophenolate mofetil or tacrolimus were initiated.

#### *Decompensated cirrhosis*

Patients with decompensated cirrhosis were initially optimized and managed according to a specific decompensating event. All patients with ascites were managed with diuretics, and those with Grade 2/3 ascites and hypoalbuminemia received albumin infusions (20 g twice/thrice a week) to control ascites. Ours is a low‐volume transplant center; hence, all patients postoptimization were offered treatment with steroids. Infections were excluded by standard criteria and cultures.^28^ In the absence of any contraindication, prednisolone was started at a dose of 0.3–0.5 mg/kg (20–30 mg) per day in patients in whom ascites could be controlled medically. Patients were closely observed for the occurrence of infections and/or other steroid‐induced adverse events. They were assessed clinically every week at the outpatient department with 2‐week liver function test (LFTs) for the initial 4 weeks. Steroids were continued at the same dose for 4 weeks. If biochemical response was present, the dose was slowly tapered by 5 mg every 2 weeks to the minimum dose required for maintenance of response. The same dose of steroids was then continued. Other immunosuppressants were not administered in decompensated cirrhosis. For patients initiated on steroids with normal liver enzymes in the presence of active disease, a close watch was kept on clinical symptoms for any deterioration. In case of no response/deterioration of clinical symptoms, liver function parameters, new‐onset/worsening existing decompensation, and/or development of signs of infection, steroids were discontinued.

### *Follow‐up*

Patients were regularly followed up on an outpatient basis as outlined above. At each visit, clinical and laboratory evaluations were performed, prognostic scores were calculated, and information on prespecified liver‐related complications (variceal bleed, new‐onset/worsening ascites, HE, acute kidney injury, and ACLF) occurring since previous visit was collected. In case of no response/deterioration of symptoms or liver functions, a decision was taken to discontinue immunosuppression, and a listing for liver transplant (LT) was carried out. In case of new‐onset acute decompensation events, patients were hospitalized and managed according to standard recommendations. Infection, when suspected, was treated with intravenous antibiotics on an inpatient basis. Patients were followed up until death or liver transplantation and were assessed for progression of disease (defined above) and for biochemical remission.

### *Study outcomes*

The primary objective was the assessment of the 12‐month transplant‐free survival rate in patients with decompensated cirrhosis stratified into different risk groups and its comparison with compensated cirrhosis. Secondary objectives were: (i) comparison of rates of biochemical remission and (ii) comparison of adverse events to treatment in different subgroups. In addition, potential predictors of outcomes (transplant‐free survival/biochemical remission) and the role of prognostic scores in the prediction of outcome in decompensated cirrhosis were evaluated.

### *Statistical methods*

The baseline data were recorded as number (%) or mean ± SD/median (interquartile range) as appropriate, based on normality of distribution. Baseline parameters were compared using chi‐square test/Fischer exact test for categorical variables and Student's *t*‐test for continuous variables with normal distribution. Continuous variables with nonnormal distribution were compared using the independent‐samples Kruskal‐Wallis test. For all statistical tests, a *P*‐value <0.05 was considered statistically significant. The 12‐month transplant‐free survival and biochemical remission rates were computed using Fine‐Gray competing risk analysis, defining three possible outcomes: biochemical remission, death, or liver transplant. Further details of competing risk analysis are outlined in Appendix S1.

Linear mixed effects models were constructed using different risk groups and times as fixed effects and patient ID as random effect for assessment of change in various biochemical parameters and prognostic indices over the first month of treatment with steroids. Results were represented using profile plots, with significance of time and different strata represented for each comparison.

Variables independently associated with survival were further assessed using a receiver‐operator characteristic (ROC) curve, and optimal cut‐offs for each predictor were identified using Youden's index. Area under the ROC (AUROC) was reported for a comparison of the accuracy of predictors.

The data were entered using Microsoft Excel 2011 and were analyzed using Rstudio. In addition to the base packages in R, ggplot2, caret, plotROC, OptimalCutpoints, Survival, survminer, cmprsk, lme4, car, dplyr, and tidyr packages were used.

## Results

**Figure 1:**
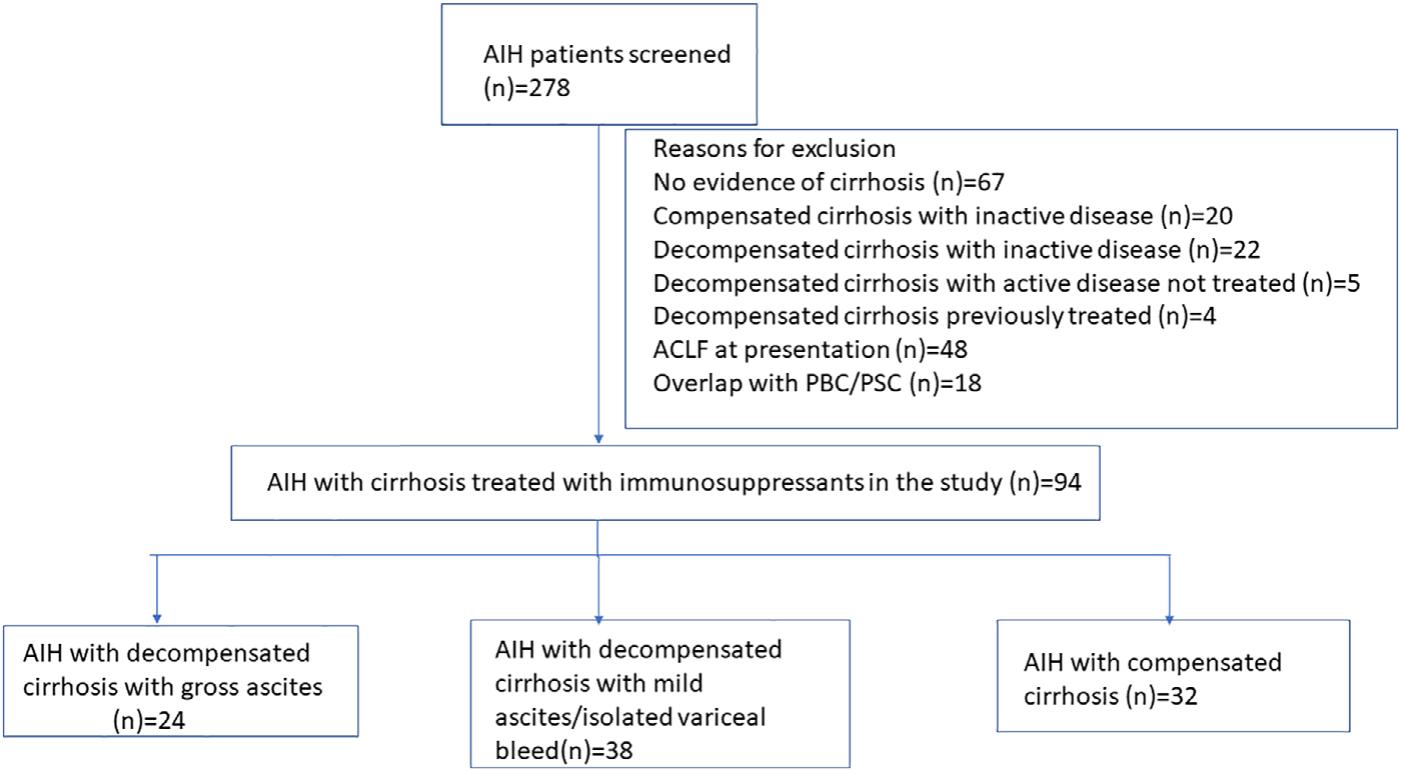
Flow diagram demonstrating number of patients examined for eligibility for inclusion in this study. Number of patients with AIH assessed (*n* = 278) and included in the study (*n* = 94) and reasons for exclusion of remaining patients.

A total of 278 patients with AIH were evaluated for the purpose of this study. Cirrhosis at presentation was seen in 189 patients (70%) patients. Among patients with cirrhosis, 45% had evidence of one or more clinical decompensation at presentation. For final analysis, 94 patients (62 decompensated and 32 compensated cirrhosis) with evidence of active disease who were started on treatment for AIH were included (Fig. 1). Thirty‐eight patients had mild ascites (Grade 1)/variceal bleed as the only decompensating event, while the remaining 24 patients had gross (Grade 2/3) ascites at presentation.

### *Baseline characteristics*

Demographic parameters such as age at presentation, duration of disease, and gender distribution were comparable among all three groups, with female preponderance among those with compensated cirrhosis and in patients with mild ascites/variceal bleed (Table 1). Among clinical presentations in the subgroup of mild/no ascites, ascites as the only decompensation event was seen in 78.9% of patients, while the remaining 21.1% patients presented with variceal bleed only. Two patients in this subgroup had both variceal bleed and mild ascites. Among patients in the gross ascites subgroup, 37.5% had more than one decompensation (ascites plus variceal bleed). Those presenting with compensated cirrhosis had a higher serum albumin level (3.5 ± 0.4 g/dL [compensated] *vs* 3.0 ± 0.5 g/dL [decompensated with mild/no ascites] and 2.9 ± 0.4 g/dL [decompensated with gross ascites], *P* = 0.001) and a lower international normalized ratio (1.2 ± 0.1 [compensated] *vs* 1.4 ± 0.3 [decompensated with mild/no ascites] and 1.6 ± 0.3 [decompensated with gross ascites], *P* = 0.001), while serum bilirubin and aminotransferase levels were comparable in all the three groups. All the patients had F4 stage of fibrosis on liver biopsy. Median HAI score on biopsy was comparable among three groups. Evidence of bile duct injury was more common in decompensated cirrhosis, being present in more than 50% of patients. Prognostic scores like CTP and MELD were significantly higher in patients with decompensated cirrhosis, being worst in the strata of decompensated cirrhosis with gross ascites at presentation.

**Table 1:** Baseline characteristics among patients with decompensated cirrhosis with gross ascites, mild/no ascites, and compensated cirrhosis

Variable	Compensated Cirrhosis (*n* = 32)	Decompensated cirrhosis with no/mild ascites (*n* = 38)	Decompensated cirrhosis with moderate/gross ascites (*n* = 24)	*P*‐value
Age (years) (mean ± SD)	36 ± 14	37.2 ± 14	39.8 ± 13.1	0.526
Females (%)	24 (75)	25 (65.8)	9 (37.5)	0.064
Duration of disease (months)	6 (4–12)	3 (1–4)	2 (1–3)	0.909
Other comorbid conditions				
Diabetes mellitus	2	0	0	0.138
Hypertension	1	0	0	0.382
IBD	1	0	0	0.382
Clinical features				
Jaundice, *n* (%)	16 (50)	25 (65.8)	13 (54.2)	0.436
Ascites, *n* (%)	0	30 (78.9)	24 (100)	<0.001
Variceal bleed, *n* (%)	0	10 (26.3)	9 (37.5)	0.001
Fatigue, *n* (%)	25 (80)	30 (78.9)	18 (75)	0.676
Asymptomatic, *n* (%)	7 (20)	0 (0)	0 (0)	0.023
Varices on EGD				<0.001
No varices, *n* (%)	18 (56.2)	4 (10.5)	2 (8.3)	
Low Grade, *n* (%)	11 (34.4)	7 (18.4)	10 (41.7)	
High grade, *n* (%)	3 (9.4)	27 (71.1)	12 (50)	
Hemoglobin (g/dL)	10.5 ± 1.6	10.2 ± 1.6	9.9 ± 1.7	0.194
WBC count (×1000/mm^3^)	6.4 ± 2.6	5.8 ± 1.6	5.2 ± 1.9	0.117
Platelets (×1000/mm^3^)	124 ± 54	86 ± 23	88 ± 33.	<0.001
Bilirubin (mg/dL)	2.1 (1.5–4.)	2.6 (1.8–5)	4 (1.7–6.6)	0.389
AST (U/L)	116 (68–194)	120 (81–170)	79 (54–180)	0.496
ALT (U/L)	102 (68–200)	94 (55–166)	75 (37–130)	0.161
ALP (U/L)	240 (180–286)	248 (162–300)	240 (183–282)	0.809
Albumin (g/dL)	3.5 ± 0.4	3 ± 0.5	2.9 ± 0.4	<0.001
INR	1.2 ± 0.1	1.4 ± 0.3	1.6 ± 0.3	<0.001
ANA titer (≥1:40), *n* (%)	23 (71.9)	21 (55.3)	14 (58.3)	0.289
ASMA titer, *n* (≥1.40) (%)	6 (18.8)	5 (13.2)	3 (12.5)	0.773
Anti‐LKM‐1, *n* (%)	2 (6.3)	1 (2.6)	1 (4.2)	0.786
IgG levels (g/dL)	2.3 ± 0.5	2.3 ± 0.6	2.4 ± 0.7	0.74
Liver biopsy				
HAI score	6 (5–7)	6 (5–8)	7 (5.5–8)	0.758
Bile duct injury, *n* (%)	11 (34.4)	25 (65.8)	14 (58.3)	0.026
CTP score	5 (5–8)	8 (7–10)	10 (9–11)	<0.001
MELD score	12.1 ± 4.6	14.7 ± 5.7	17.7 ± 5.5	0.006
Child‐Pugh class				<0.001
Class‐A, *n* (%)	19 (59.4)	6 (15.8)	0 (0)	
Class‐B, *n* (%)	13 (40.6)	20 (52.6)	9 (37.5)	
Class‐C, *n* (%)	0	12 (31.6)	15 (62.5)	

Data are presented as mean ± SD or median (interquartile range) for quantitative variables and *n* (%) for qualitative variables unless otherwise specified.

ALP, alkaline phosphatase; ALT, alanine transaminase; ANA, antinuclear antibody; ASMA, anti‐smooth muscle antibody; AST, aspartate transaminase; CTP, Child Turcot Pugh; EGD, esophago‐gastroduodenoscopy; HAI, histological activity index; IBD, inflammatory bowel disease; IgG, immunoglobulin G; INR, international normalized ratio; LKM‐1, liver‐kidney microsome; MELD, model for end‐stage liver disease; SD, standard deviation; WBC, white blood cell.

### *Prednisolone dosage and duration in the strata*

The median daily dose of steroid used was 25 mg (20–30 mg) in the subgroup of decompensated cirrhosis with mild/no ascites and 20 mg (20–25 mg) in patients with gross ascites (Table 2). This dose was lower in comparison to that used in compensated disease (40 mg [30–50 mg]; *P* < 0.01). Overall, 44 patients (72.4%) with decompensated cirrhosis required discontinuation of steroids, with no biochemical response (*n* = 13), progression of liver disease while on steroids (*n* = 11), and development of infections (*n* = 21) being the main indications. Almost all patients with gross ascites (95.8%) and 57.8% of patients with mild/no ascites required steroid discontinuation. After 6 months of treatment, only 15% of patients with decompensated cirrhosis were maintained on prednisolone, and a majority of them were on doses lower than 10 mg/day.

**Table 2:** Comparison of outcomes among patients with decompensated cirrhosis with gross ascites, mild/no ascites, and compensated cirrhosis

Variable	Compensated cirrhosis (*n* = 32)	Decompensated cirrhosis with no/mild ascites (*n* = 38)	Decompensated cirrhosis with moderate/gross ascites (*n* = 24)	*P*‐value
Immunosuppressants				0.072
Steroids	32 (100)	38 (100)	24 (100)	
Azathioprine	24 (75)	0	0	
MMF	4 (12.5)	0	0	
Tacrolimus	1 (3.1)	0	0	
Dose of prednisolone (mg/day)	40 (30–50)	25 (20–30)	20 (20–25)	0.01
Discontinuation of steroids	1 (3.1)	21 (57.8)	23 (95.8)	0.001
Clinical outcome				<0.001
No change	5 (15.6)	15 (39.5)	9 (37.5)	
New decompensation	2 (6.2)	3 (7.9)	2 (8.3)	
Worsening of ascites	0	3 (7.9)	3 (12.5))	
ACLF	0	2 (5.3)	8 (33.3)	
Adverse effects				
Infection	3 (9.3)	9 (23.7)	12 (50.0)	0.002
Leukopenia	8 (25)	0	0	<0.001
Diabetes	8 (25)	12 (31.5)	6 (25)	0.782
Hypertension	2 (6.2)	0	0	0.139
Rise in IOP	1 (3.1)	0	0	0.381
Duration of follow‐up (months)	12 (7–28)	5 (4–9)	3 (2–6)	<0.001
Biochemical remission	17 (53.1)	12 (31.6)	1 (4.2)	0.001
Deaths	0	6 (15.8)	16 (66.7)	<0.001
Liver transplant	1 (3.1)	3 (7.9)	1 (4.2)	0.41

Data are presented as mean ± standard deviation or median (interquartile range) for quantitative variables and *n* (%) for qualitative variables unless otherwise specified.

ACLF, acute‐on‐chronic liver failure; IOP, intraocular pressure; MMF, mycophenolate mofetil.

### *Treatment outcomes*

#### *Survival*

**Figure 2:**
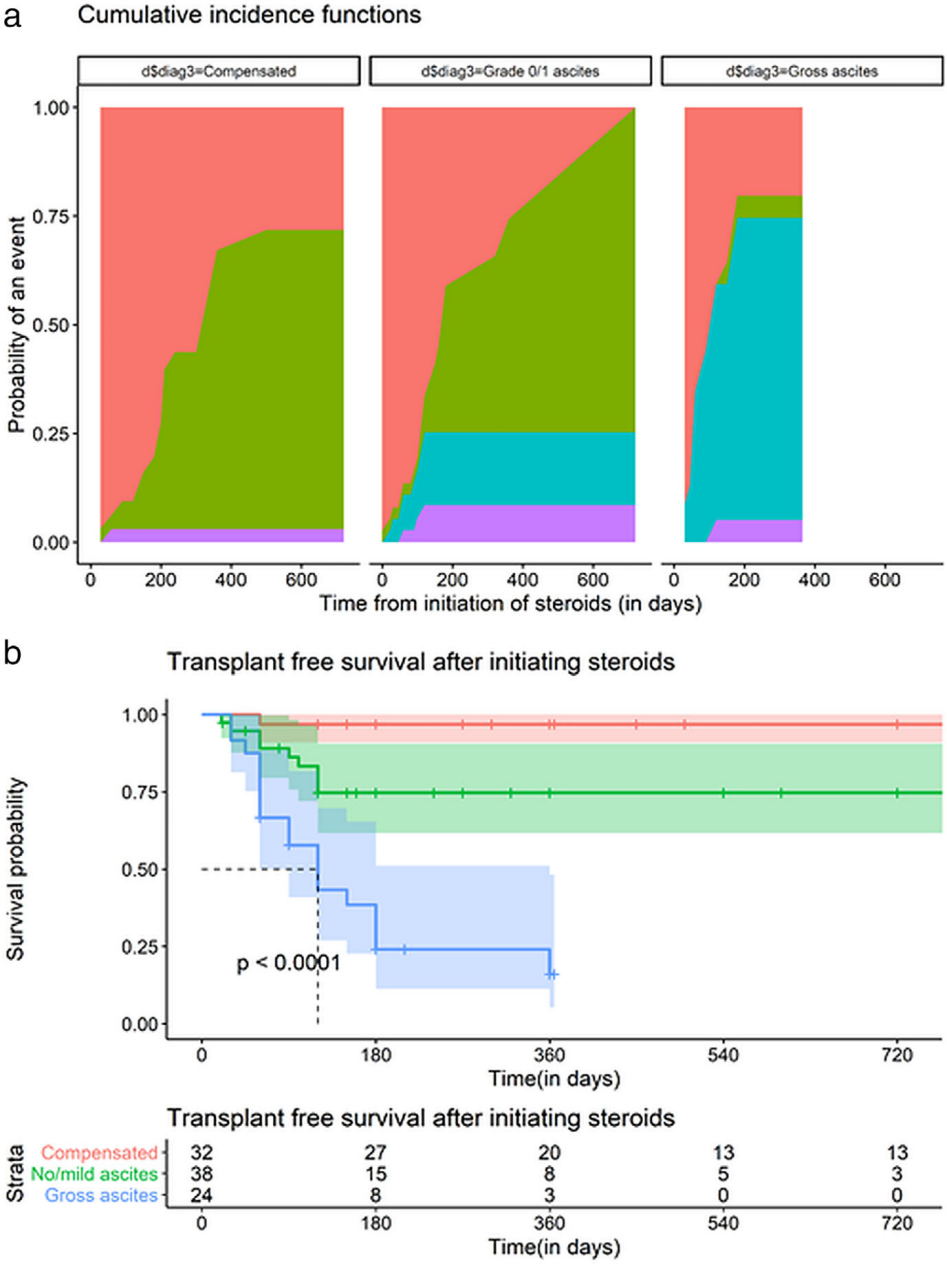
Outcomes on treatment with steroids in autoimmune hepatitis (AIH)‐related cirrhosis in the strata of compensated cirrhosis, decompensated cirrhosis with mild/no ascites, and decompensated cirrhosis with gross ascites. (a) Competing risks plot demonstrating rates of biochemical remission, death, and liver transplant over duration of follow‐up in all three strata of AIH‐related cirrhosis. 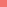, Event; 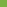, biochemical remission; 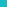, death; 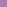, liver transplant. (b) Kaplan–Meier plot of transplant‐free survival in same population. 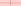, Compensated; 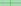, no/mild ascites; 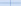, gross ascites.

Overall, 16 (66.7%) patients with gross ascites and 6 (15.8%) patients with mild/no ascites died over the follow‐up period, whereas none in compensated cirrhosis died (Table 2; Fig. 2a,b). In addition, one patient with gross ascites (4.2%) and three (7.9%) patients with mild ascites/no ascites underwent LT over the follow‐up period. One patient in compensated cirrhosis also underwent LT in view of progressive decompensation. The 12‐month transplant‐free survival, taking LT as a competing event, was 30%, 74.5%, and 96.8% in those with gross ascites, mild/no ascites, and compensated cirrhosis, respectively (Gray's test, *P* = 0.001 for comparison between all three strata; Gray's test, *P* = 0.016 for comparison between compensated cirrhosis and mild/no ascites).

#### *Biochemical remission*

Twelve patients (31.6%) in the mild/no ascites and 1 (4.2%) in the gross ascites groups attained biochemical remission over the follow‐up period, whereas 17 patients (53.1%) with compensated cirrhosis attained the above end‐point (Fig. 2a). The 12‐month biochemical remission rates, taking mortality and LT as the competing events, were 5%, 49%, and 64% in patients with gross ascites, mild/no ascites, and compensated cirrhosis, respectively (Gray's test, *P* = 0.001 for comparison between all three strata, Gray's test, *P* = 0.9 for comparison between compensated cirrhosis and mild/no ascites).

#### *Infections and other adverse events*

Infections and other events are described in detail in Appendix S1 and in Table 2.

#### *Other events*

Other events and further complications are summarized in Appendix S1 and in Table 2.

#### *Predictors of response to steroids in decompensated cirrhosis*

Predictors of response to steroids in decompensated cirrhosis are outlined in Appendix S1 and in Table 3.

**Table 3:** Multivariable adjusted and corresponding univariate analyses for two major posttreatment outcomes. Transplant‐free survival and biochemical remission were assessed using multivariate Fine‐Gray subdistributional hazards model

	Univariate Fine‐Gray subdistributional hazards ratio (95%CI)	*P*‐value	Multivariate Fine‐Gray subdistributional hazards ratio (95%CI)	*P*‐value
Transplant‐free survival				
Biochemical remission	0.112 (0.0165–0.765)	0.026	0.295 (0.0313–2.78)	0.29
MELD score	1.17 (1.1–1.25)	0.001	1.153 (1.07–1.24)	0.001
Infection	4.24 (1.82–9.83)	0.001	2.222 (0.892–5.54)	0.086
Ascites grade 2/3	4.37 (2.0–9.56)	0.001	2.556 (1.565–5.65)	0.020
Biochemical remission				
Serum albumin	2.84 (1.57–5.13)	0.001	2.699 (1.145–6.359)	0.023
MELD score	0.923 (0.864–0.987)	0.019	1.002 (0.9214–1.09)	0.96
Ascites grade 2/3	1.86 (0.926–3.75)	0.081	0.684 (0.299–1.561)	0.37
Infection	0.090 (0.011–0.686)	0.022	0.107 (0.012–0.916)	0.041

MELD, model for end‐stage liver disease.

#### *Laboratory changes and immediate treatment response across different risks strata*

**Figure 3:**
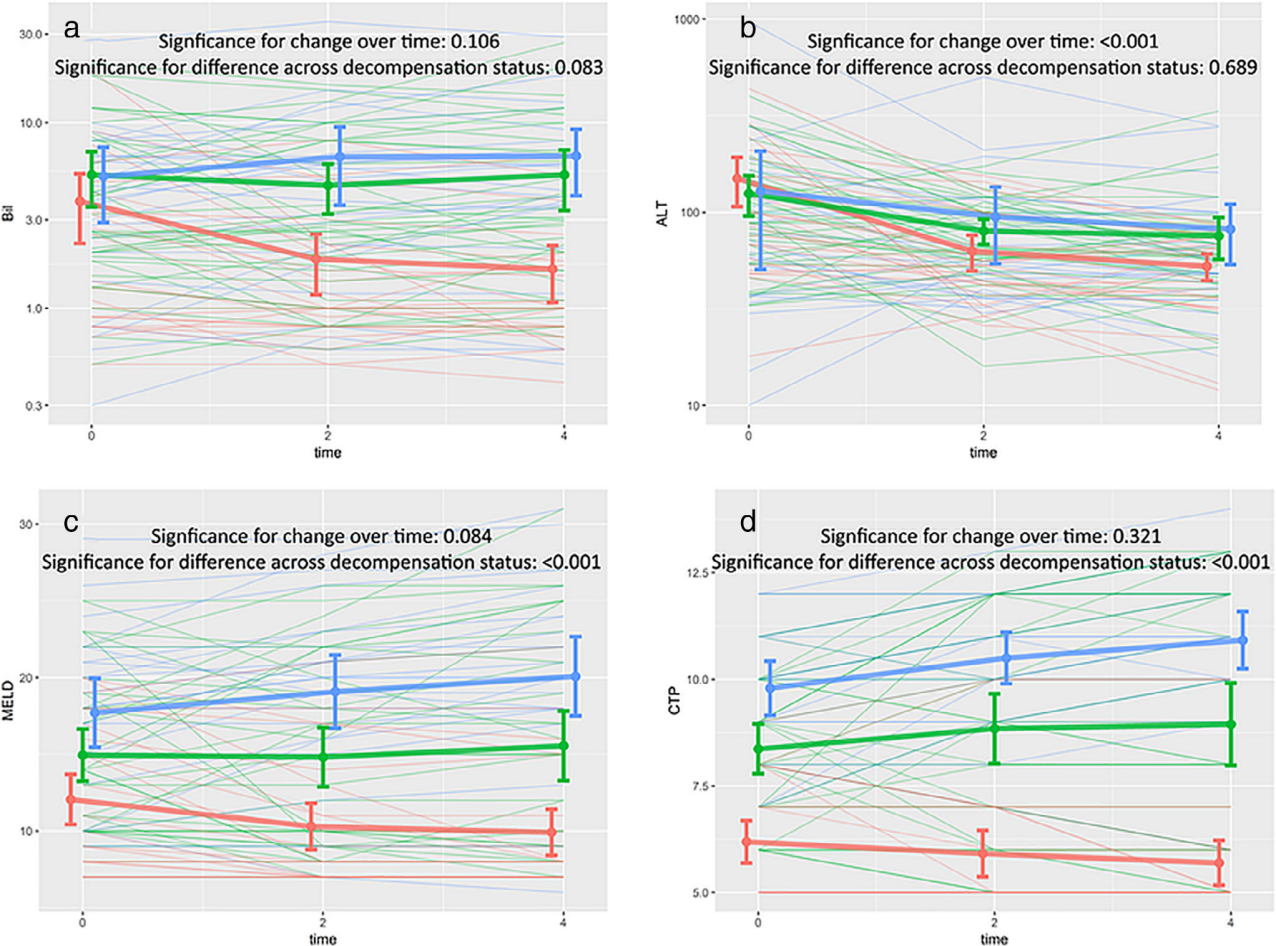
Profile plots demonstrating change in (a) serum bilirubin, (b) serum alanine transaminase (ALT), (c) model for end‐stage liver disease (MELD), and (d) Child Turcot Pugh (CTP) scores over first month of treatment with steroids, in all the three strata at treatment initiation. Linear mixed effects models were used to compute significance of change across different strata and over time, shown with respective plots. Colors represent different strata, with thicker lines representing means of respective strata. Error bars represent standard deviation at that particular time point. Time on x‐axis is in weeks from initiation of treatment. 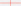, Compensated; 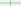, no/mild ascites; 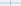, gross ascites.

Changes in biochemical parameters and treatment response are detailed in Appendix S1 and Figure 3.

#### *Performance of different scores/indices in predicting outcome on steroids*

**Figure 4:**
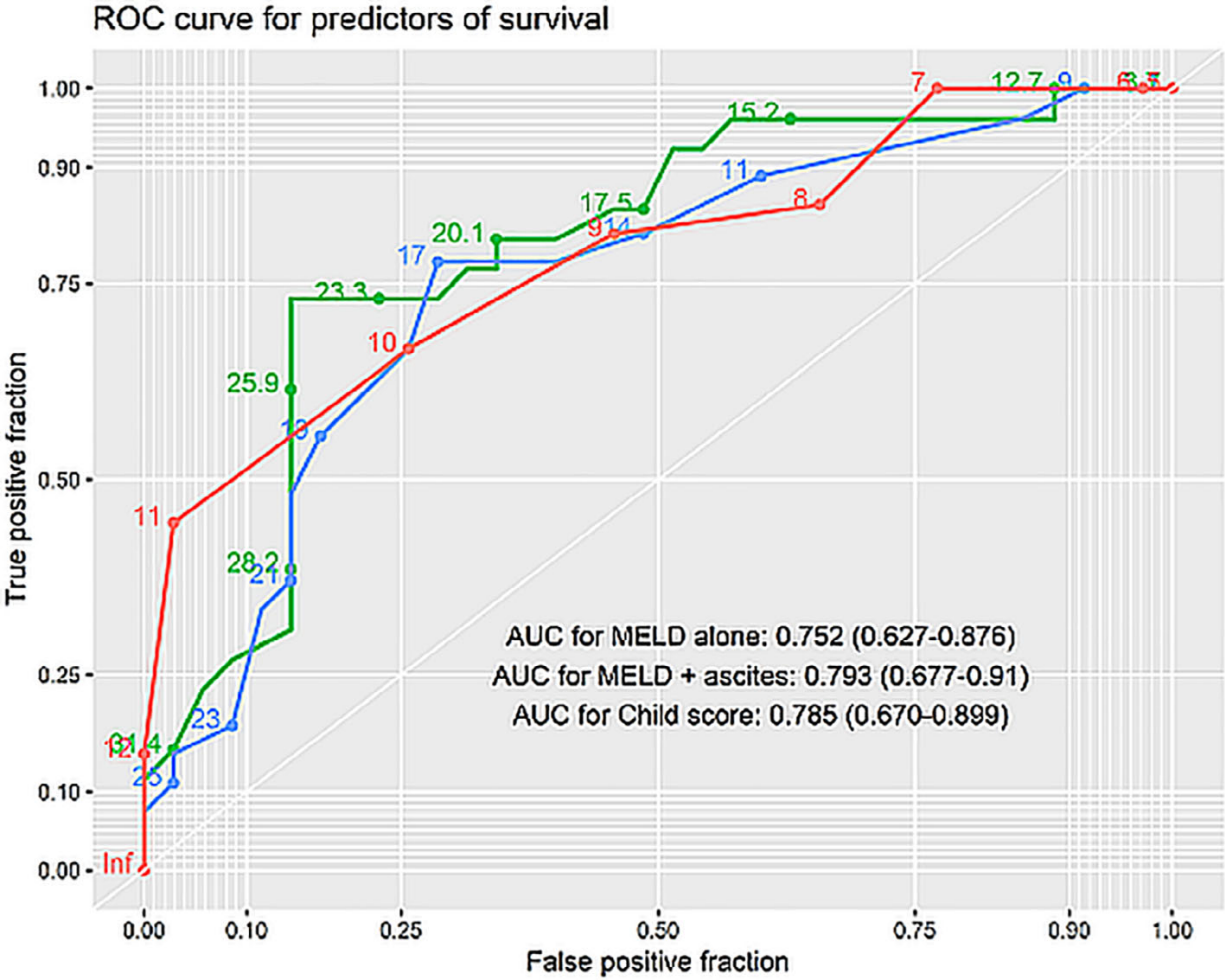
Receiver operator characteristics (ROC) curves for predictors of survival. Numbers on plot represent value of respective prognostic variable. Area under curve for different indices are indicated in the plot along with their 95% confidence intervals. 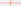, Child score; 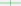, model for end‐stage liver disease (MELD) + ascites; 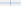, MELD alone.

The prognostic performance of different scores is outlined in Appendix S1 and Figure 4.

## Discussion

In the present study, patients with AIH with decompensated disease at presentation had poorer outcomes on steroids when compared to those with compensated disease. More than 70% of patients required discontinuation of the drug due to a lack of response/adverse events. The dosage of steroids given in decompensated disease was lower than that of compensated cirrhosis. Among the patients with decompensated cirrhosis, those with mild ascites and/or variceal bleed as the only decompensation had relatively good outcomes with comparable incidence of biochemical remission and adverse events as in compensated cirrhosis. Baseline severity of disease remained the most important predictor of overall outcome.

In our study, 70% patients of the whole cohort had cirrhosis at presentation, with 45% of these patients having clinical evidence of decompensation. This proportion of cirrhosis at presentation is similar to previously described cohorts of AIH from this region and is higher than those described from the West.^2^, ^3^, ^9^, ^10^, ^18^, ^20^, ^29^ In addition, direct presentation with decompensated cirrhosis is uncommonly described from western cohorts. Late presentation, referral bias, and/or aggressive behavior of disease are the possible reasons for higher frequency of decompensated cirrhosis reported in this region.

Among clinical decompensations, gross ascites at presentation was associated with poor response to steroids and decreased survival when compared to those patients who had only mild ascites and/or variceal bleed as the only decompensation. Treatment of ongoing inflammation/disease activity holds little relevance in patients who have decompensated with clinical ascites. Despite a reduction in aminotransferase levels on treatment across all strata, MELD and CTP scores worsened in patients who had gross ascites at baseline. Overall, patients with a MELD score of more than 17 had poor outcomes, with a limited role of immunosuppression. Liver transplant may be the only definitive option in patients with clinical ascites and with a higher MELD score.

Patients presenting with isolated variceal bleed/mild ascites, when treated, had a higher transplant‐free survival rate compared with those with gross ascites. While survival in this subgroup was lower than those with compensated cirrhosis, attainment of biochemical remission and incidence of posttreatment infections were comparable in both these groups. The findings from the present study suggest that the treatment of underlying ongoing inflammation with low‐dose steroids in this subgroup may be beneficial.

Among adverse events, bacterial infections developed more frequently in patients with decompensated cirrhosis with gross ascites, while the incidence of infections in mild/no ascites was comparable to those with compensated cirrhosis. Overall, patients with posttreatment infections had decreased transplant‐free survival. The frequency of other adverse events was comparable among patients with and without clinical decompensations.

Fewer studies in the past had evaluated outcomes of steroids in patients with decompensated cirrhosis. Wang *et al*. demonstrated the reversal of clinical decompensations in 62% of patients with limited information on other treatment‐related outcomes, such as biochemical remission.^19^ Amarapurkar *et al*., in their study, demonstrated biochemical remission in 68% of these patients,^18^ a rate much higher than the present study. Notable differences with the present study included the lack of histological confirmation and detailed algorithm in the management of these patients. We believe that more studies with a longer follow‐up are required to conclusively address the role of steroids in this condition.

There are certain important limitations in this study. First, in the absence of a control group, the survival benefit of steroids demonstrated in this study needs further validation with a comparison with controls not treated with steroids. In the present cohort, patients with decompensated cirrhosis not treated with steroids had a more severe liver disease and/or contraindication to steroids at presentation and, therefore, could not be used as controls for the treated subgroup. However, comparable rates of biochemical remission (which is otherwise rare in AIH spontaneously^30^) in the strata of mild/no ascites and those with compensated cirrhosis suggest a potential role of steroids in the management of decompensated cirrhosis. Second, patients with decompensated cirrhosis had a limited period of follow‐up duration. From our study, long‐term outcomes in terms of overall survival and relapse of disease cannot be deduced.

In conclusion, our study showed that type and severity of clinical decompensations primarily affect outcomes in patients with AIH‐related cirrhosis when administered low‐dose steroids. Patients with mild ascites/variceal bleed achieve comparable rates of biochemical remission compared to those with compensated disease. Such patients may be administered low‐dose steroids to improve the outcome. However, in the absence of survival benefit, long‐term outcomes remain undefined in these patients and need to be analyzed in future studies.

## Supporting information


**Appendix** **S1**. Methodology.
