# Supplementary material for: Presence and type of decompensation affects outcomes in autoimmune hepatitis upon treatment with corticosteroids
Source: JGH Open. 2020 Nov 13;5(1):81–90. doi: 10.1002/jgh3.12451 (PMC7812520; doi:10.1002/jgh3.12451)
Supplement: Supplementary file 1 — Appendix S1. Methodology. [file JGH3-5-81-s001.docx]

**SUPPLEMENTARY CONTENT**

| **Content** | **Page** |
| --- | --- |
| **Methodolgy** | **2** |
| **Statistical analysis** | **3** |
| **Results** | **4** |

**Methodology**

*Data collection*

The demographic profile, clinical records, laboratory data, liver biopsies, endoscopic/imaging data and outcomes of these patients upon treatment were reviewed from a prospectively maintained database. Upper gastrointestinal endoscopy for variceal screening was done in every patient. Laboratory data including serological data [ANA (via indirect immunofluorescence) Anti LKM1 (via ELISA), and ASMA (via indirect immunofluorescence)] and serum IgG levels were reviewed in included patients. Additional antibodies specific for AIH were not done. Liver biopsies were done either percutaneously (in patients with no ascites/coagulopathy) or via transjugular route (in remaining patients). The liver biopsies were reviewed for HAI, stage of fibrosis, evidence of bile duct injury and canalicular/cytoplasmic cholestasis (if present). Efficacy outcomes (biochemical remission, clinical remission and survival) and safety outcomes (adverse events) were retrieved from the database.

**Statistical analysis**

Competing risk analysis

Survival was compared using Gray’s test, with competing-risks plot and Kaplan-Meier plot to represent survival across strata. For outcomes where time to event data was available (transplant-free survival and biochemical remission), Fine and Gray subdistribution hazards models were constructed using liver transplant as competing risk for survival and both death as well as liver transplant as competing risks for biochemical remission. Variables that were significantly different on univariate analysis between those who attained these outcomes vis-à-vis those who did not were considered for inclusion in the multivariate model. Variables measuring similar characteristics were excluded to avoid multicollinearity, and those with limited clinical significance were not included to maintain event per variable rate less than 5, thus preventing unstable estimates. Results were expressed as subdistribution Hazards ratio (sHR) with 95% confidence intervals and p-values for both univariate and multivariate analyses. Clinical improvement was assessed using generalized linear model with logit link function, binomial response, 5-fold cross-validation and 3-fold repetition, with variable inclusion as for subdistribution hazards model. Results were reported as odds ratio (exponent of coefficients) with 95% confidence intervals and significance for both univariate and multivariate analysis

**Results**

*Infections and other adverse events*

Infections developed in 12(50%), 9(23.7%) and 3(9.3%) patients with gross ascites, mild/no ascites and compensated cirrhosis respectively (p=0.002 for comparison between three groups, p=0.102 for comparison between compensated cirrhosis and those with mild/no ascites). Among site of infections, Spontaneous bacterial peritonitis (SBP) was the most common infection in decompensated group [11 (52.9%) patients], followed by pneumonia [7 (33.3%) patients] and urinary tract infection (UTI) [3 (14.8%) patients]. In compensated group, UTI was the only infection noted [3 patients].

Among other adverse events, leukopenia developed in one third of patients with compensated cirrhosis who received azathioprine. Steroid induced diabetes developed in 25% patients with compensated cirrhosis, 31.1% patients with mild/no ascites and in 25% patients with gross ascites. Other complications like rise in intra-ocular pressure and hypertension were exclusively seen in compensated cirrhosis.

*Other events (Table-2)*

New decompensation events occurred in 5 patients while ascites worsened in 6 patients with decompensated cirrhosis. Among new decompensation events, one patient with no previous ascites developed ascites while 4 patients developed HE. Two patients with compensated cirrhosis developed decompensation (1 with variceal bleed and another with ascites) over the period of follow-up. Acute on chronic liver failure developed in 10 patients with decompensated cirrhosis (2 patients in mild/no ascites and 8 patients in gross ascites).

*Predictors of response to steroids in decompensated cirrhosis*

Transplant-free survival: Attainment of biochemical remission, baseline MELD score, development of post-treatment infection and presence of grade-2/3 ascites were included in the multivariate subdistribution hazards model. On multivariate analysis, only presence of Grade-2/3 ascites [subdistribution Hazards Ratio[sHR]: 2.556 (95% confidence interval: 1.565-5.65); p=0.020] and MELD score [sHR: 1.153 (1.07 - 1.24); p=0.001] independently predicted survival.

Biochemical remission: Baseline serum albumin, development of infection post treatment and MELD score were significantly associated with biochemical remission on univariate analysis, and were included in the multivariate model, along with ascites. Serum albumin levels [sHR: 2.699(1.145-6.359); p=0.023] and development of post-treatment infections [sHR: 0.107 (0.012-0.916); p=0.041] independently predicted biochemical remission.

*Laboratory changes and immediate treatment response across different risks strata*

Changes in serum bilirubin, ALT, MELD and CTP score were evaluated at week 0,2 and 4 post initiation of steroids and was stratified into the previously outlined risk groups class (Figure-3). While there was a significant decline in ALT levels over the period of first month across all the strata, those with gross ascites had worsening of prognostic scores (MELD score and CTP score) with steroids when compared with other two strata.

*Performance of different scores/indices in predicting outcome on steroids*

Performance of different scores for predicting survival in steroid treated decompensated cirrhotics was assessed by plotting ROC curves for CTP and MELD (Figure-4). In addition, ascites was incorporated in MELD score based on its independent significance noted in multivariable adjusted model. The AUROC for CTP score and MELD score for predicting survival was 0.785(0.670-0.899) and 0.752(0.627-0.876) respectively. A cut-off of MELD score of 17 had 86(65-97) % sensitivity and 70(53-83) % specificity for predicting mortality. Child-Turcot-Pugh score of 10 had a sensitivity of 72(49-89) % and specificity of 72(56-85) % for predicting mortality. Addition of ascites to MELD score marginally increased its AUROC to 0.793 (0.677-0.91).
